# Detection of severe hypertension in a patient with neurofibromatosis type 1 during anesthesia induction: a case report

**DOI:** 10.1186/s13256-019-2292-4

**Published:** 2019-11-30

**Authors:** Juan Wang, Guohua Wei, Zhongyun Wang, He Huang

**Affiliations:** Department of Anesthesiology and Perioperative Medicine, First Affiliated Hospital of Nanjing Medical University, Nanjing, China

**Keywords:** Pheochromocytoma and paraganglioma, Neurofibromatosis type 1, Screening, Catecholamine

## Abstract

**Background:**

Neurofibromatosis type 1 has a higher prevalence of pheochromocytoma and paraganglioma than the general population: 1.0–5.7% versus 0.2–0.6%. Currently, there are no generally accepted guidelines for screening for pheochromocytoma and paragangliomas in asymptomatic patients with neurofibromatosis type 1.

**Case presentation:**

Severe hypertension developed during anesthesia induction in our patient, a 44-year-old Chinese man with neurofibromatosis type 1. We screened for catecholamine level after glioma resection, and the patient was diagnosed with combined pheochromocytoma and paraganglioma.

**Conclusions:**

A delay in diagnosis or lack of a diagnosis in pheochromocytoma and paraganglioma may increase the perioperative morbidity and mortality risk due to excess catecholamine secretion. Therefore, routine pheochromocytoma and paraganglioma screening preoperatively in patients with neurofibromatosis type 1 is very important.

## Background

Neurofibromatosis (NF) is a complex, multisystem, autosomal dominant disease that has widespread effects on ectodermal and mesodermal tissue. Approximately 6% of cases of neurofibromatosis type 1 (NF1) manifest hypertension, which may be highly associated with renovascular disease, renal artery stenosis, and pheochromocytoma and paraganglioma (PPGL) [1]. Previous studies suggested that patients with NF1 had an increased prevalence of PPGL (1.0–5.7%) compared with patients with hypertension (0.2–0.6%) in an outpatient clinic survey [2, 3]. Therefore, undiagnosed PPGL or delayed PPGL diagnosis in normotensive and asymptomatic patients may contribute to significant perioperative morbidity and mortality risk due to excess catecholamine secretion. Currently, there are no universally accepted guidelines for screening and detection of PPGL in individuals with NF1 lacking catecholamine-associated symptoms and/or hypertension. Recent literature suggested that patients with NF1 could benefit from routine screening for PPGL perioperatively [4]. In this report, we present a case involving screening for PPGL in a patient with NF1 after glioma resection.

## Case presentation

A 44-year-old Chinese man, weight 55 kg and height 165 cm, presented to the neurosurgery department of our institution with an 11-month history of paroxysmal headache and weakness of the right limb without any other pertinent positive symptom. The patient had been diagnosed with NF1 for more than 30 years. His past surgical history included having undergone left parietal tumor resection in May 2016, the postoperative pathology of which indicated glial sarcoma (World Health Organization grade IV). In July 2017, the patient presented with paroxysmal headache with weakness of the right limb. Computed tomography (CT) of the head revealed recurrence of left parietal glioma, acute cerebral infarction in the left frontal lobe and around ventricle. As a result, the patient received conservative treatment in the neurology department.

**Fig. 1:**
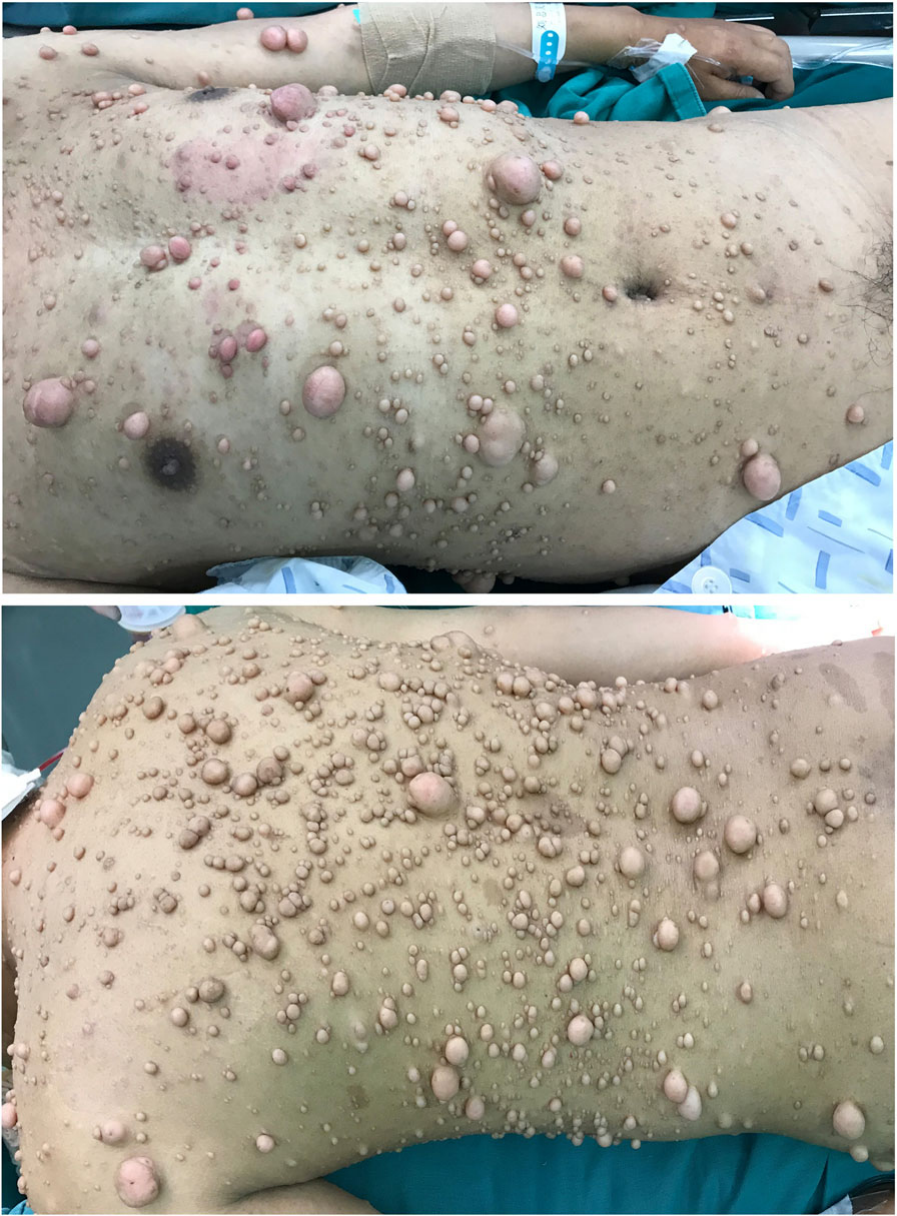
Multiple cutaneous neurofibromata and numerous café-au-lait spots dispersed over the trunk and limbs

The only pertinent positive physical examination finding was multiple cutaneous neurofibromas and numerous café-au-lait spots of different sizes dispersed over the trunk and limbs (Fig. 1). The results of the patient’s cardiovascular, respiratory, and abdominal examinations were all unremarkable. His baseline blood pressure was in the range of 120–140 mmHg for systolic blood pressure and 65–85 mmHg for diastolic blood pressure. He denied any current medication, tobacco, or alcohol use, and a family history of NF was also excluded.

The results of the patient’s preoperative laboratory examination were unremarkable, including blood and urine analysis. His electrocardiographic examination showed normal sinus rhythm. His chest x-ray revealed multiple nodules in the two lung fields, the largest being located in the upper right quadrant of the lung field and having a diameter of about 29 mm. Magnetic resonance imaging of the head revealed the recurrence of glioma. The patient was scheduled for resection of the recurrent gliomas.

During the preoperative examination, it was thought that general anesthesia application would be more appropriate for the patient. The patient was monitored with electrocardiography, heart rate (HR), invasive blood pressure, and pulse oximetry in the operating room. He was anesthetized with midazolam 3 mg, etomidate 14 mg, cisatracurium 20 mg, fentanyl 0.15 mg, and propofol 60 mg in sequence. When assisted respiration was started, the monitor showed a persistent increase in blood pressure. Within 40 seconds, it rose to about 310/140 mmHg, and the HR increased to about 140 beats per minute (bpm). We quickly eliminated the following possibilities: taking the wrong medicine, blood pressure monitoring equipment malfunction, or problem with venous access. To prevent the cardiovascular complications, we took measures to control the patient’s blood pressure and HR with phentolamine 2 mg, esmolol 30 mg, and remifentanil 80 μg when the blood pressure was about 310/140 mmHg and HR was about 140 bpm. The blood pressure values were stable during intubation, but the HR continued to be higher than 110 bpm.

The patient’s blood pressure was stable during the operation. However, there was a fluctuation of blood pressure during extubation with an increase to as high as 210/140 mmHg, which was aborted with phentolamine 1 mg. Nevertheless, the patient’s HR continued to be higher than 120 bpm, and he was not sensitive to β-adrenergic blockade.

**Fig. 2:**
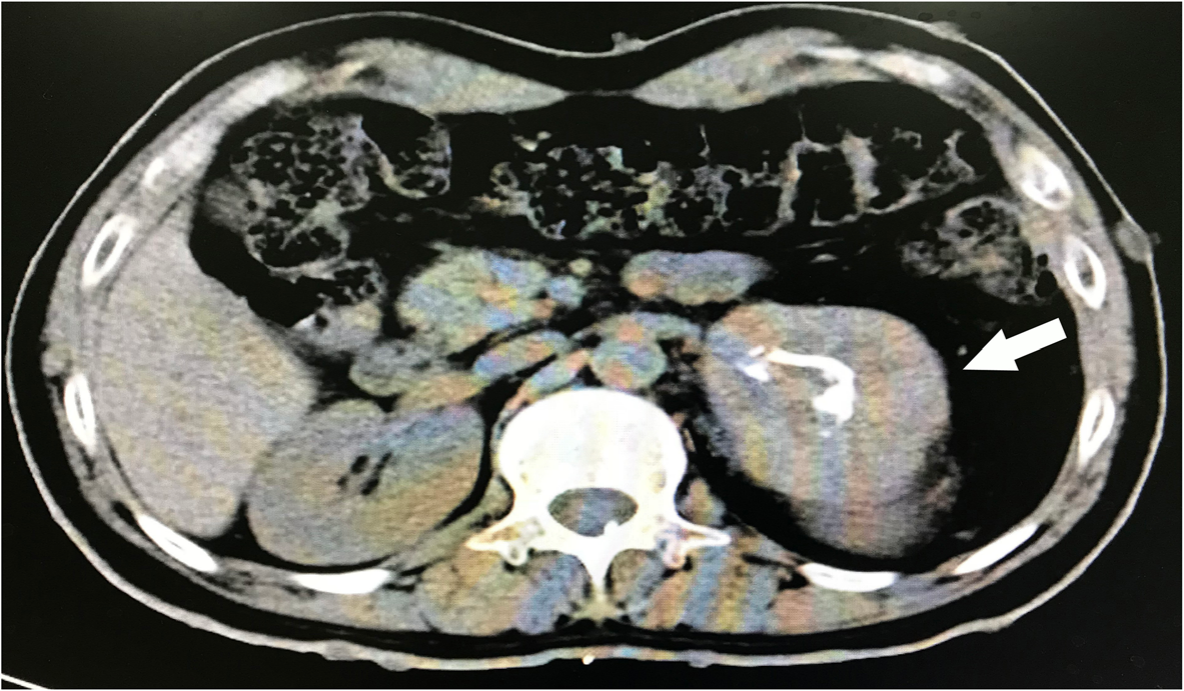
Enhanced CT of abdomen demonstrated a 7.7 cm × 6.7cm heterogeneous mass in the left adrenal gland (white arrow)

During postoperative follow-up, no headache, nausea, or blood pressure change (especially hypotension) was observed, and the patient’s tachycardia disappeared 3 days after the operation. To investigate the causes of severe hypertension during anesthesia induction, we initiated a biochemical workup of his adrenal hormone 3 days after the operation, which revealed elevated 24-hour blood laboratory test results: epinephrine 3.57 nmol/L (normal range, 0.01-0.34 nmol/L), metanephrine 8.99 nmol/L (0.01–0.42), normetanephrine 2.25 nmol/L (0.01–0.71), and vanillylmandelate 160.41 nmol/L (0.01–62). Subsequently, CT of the abdomen with contrast enhancement (Fig. 2) demonstrated a 7.7-cm × 6.7-cm heterogeneous mass in the left adrenal gland.

## Discussion

NF1 is an autosomal dominant disorder characterized by a tendency to form tumors of ectodermal and mesodermal tissues. Clinical diagnosis is based on two or more of the following National Institutes of Health diagnostic criteria: café au lait macules, neurofibromas, axillary/inguinal freckling, Lisch nodules, distinctive osseous lesions, glioma, and/or family history of NF [5].

The anesthetic management of patients with NF1 requires careful systemic preoperative evaluation, including airway management, respiratory and cardiovascular complications, central nervous system involvement, and vertebral anomalies. Hypertension should be examined because of the 5.7–14% prevalence of PPGL in patients with NF1. Literature has suggested that the prevalence of PPGL in patients with NF1 is higher than in the general population [6, 7]. Due to the lack of general consensus on PPGL case detection in the asymptomatic and normotensive NF1 population, these patients have the potential for excess catecholamine secretion in the perioperative period, which can lead to elevated cardiovascular morbidity and mortality.

Our patient with NF1 had a normal heart rhythm and blood pressure preoperatively; however, he had significant catecholamine-associated symptoms during anesthesia induction and the perioperative period. The postoperative blood laboratory test results showed elevated catecholamine production, and abdominal CT with contrast enhancement revealed a large mass in the left adrenal gland. These biochemical and imaging examinations suggested that our patient had NF1 combined with PPGL. Recent literature has suggested that 24% of patients with NF1 with PPGL will remain without catecholamine-associated symptoms and that 61–80% will not have hypertension, despite having similarly elevated plasma and urine metanephrine concentrations compared with symptomatic patients [4]. Therefore, after diagnosis of NF1, patients who have episodes of hypertension (the most consistent clinical sign), sweating, headache, and palpitation should be evaluated for pheochromocytoma preoperatively, and anesthesiologists must maintain a high index of suspicion for the possibility of pheochromocytoma. It would be suitable to start treatment with an α-blocker (phenoxybenzamine) when preoperative hypertension is detected, and preparation for α-blockers and β-blockers is essential. Although there were no cardiovascular complications in our patient, it should not be forgotten that an undiagnosed or disregarded pheochromocytoma may lead to an intraoperative life-threatening hypertensive crisis in patients with NF [8].

## Conclusion

Although the prevalence of PPGL in patients with NF1 is higher than in the general population, a majority of patients remain asymptomatic and normotensive. Currently, there are no generally accepted clinical practice guidelines for screening asymptomatic patients with NF1; therefore, we highlight that routine PPGL screening may have benefit in patients with NF1 by providing an earlier diagnosis, avoiding perioperative catecholamine-associated symptoms, and preventing increase of morbidity and mortality risks.

## Data Availability

Not applicable.
